# Gastric Necrosis and Perforation Secondary to Severe Diabetic Gastroparesis in a Young Woman With Type 1 Diabetes Mellitus: A Case Report

**DOI:** 10.7759/cureus.111798

**Published:** 2026-06-30

**Authors:** Marina Skovronski, Luiz Augusto Sousa Oliveira, Luís Henrique Zahner, Camilo Tupac Dias Pirez, Leonardo José Urmann, Fernando Savegnago

**Affiliations:** 1 General Surgery, Fundação Hospitalar Santa Terezinha de Erechim, Erechim, BRA; 2 Intensive Care Unit, Fundação Hospitalar Santa Terezinha de Erechim, Erechim, BRA

**Keywords:** acute gastric dilatation, case report, diabetic gastroparesis, diabetic ketoacidosis, gastric necrosis, gastric perforation, type 1 diabetes mellitus

## Abstract

Diabetic gastroparesis is a common chronic complication of diabetes mellitus, usually manifesting as nausea, vomiting, early satiety, and impaired glycemic control. Its progression to acute gastric dilatation with ischemic necrosis and perforation is exceptionally rare but carries a very high mortality. We report the case of a 19-year-old woman with poorly controlled type 1 diabetes mellitus and recurrent diabetic ketoacidosis (DKA) who developed gastric necrosis and perforation presumptively attributable to severe diabetic gastroparesis. Over approximately four months, she experienced progressive epigastric pain, recurrent vomiting, and weight loss. Serial computed tomography (CT) documented the evolution: an initially unremarkable stomach, followed by marked gastric and duodenal distension with an antral air-fluid level, and finally pneumoperitoneum with abundant free intraperitoneal fluid, with perforation confirmed at laparotomy. She presented critically ill with severe DKA (pH 6.87, glucose 1002 mg/dL) and septic shock. Emergency exploratory laparotomy disclosed approximately 3.5 L of gastrobiliopancreatic fluid and food debris, with ischemic necrosis of the gastric fundus and multiple perforations (the largest measuring 1.5 cm). A partial (fundic) gastrectomy with two-layer gastrorrhaphy was performed. The postoperative course was marked by septic shock and severe malnutrition (albumin 1.8 g/dL). This case illustrates that severe diabetic gastroparesis can, although rarely, culminate in gastric ischemia and perforation, a surgical emergency, and highlights the value of serial imaging, aggressive glycemic and nutritional management, and timely surgical source control. With aggressive resuscitation, source control, broad-spectrum antimicrobial therapy, and structured nutritional support, the patient improved; she was extubated, weaned from vasopressors, and progressed to enteral nutrition, with a favorable prognosis at the most recent assessment.

## Introduction

Gastroparesis is a chronic disorder defined by objectively delayed gastric emptying in the absence of mechanical obstruction, and diabetes mellitus is its most common identifiable cause [1,2]. Its pathophysiology is multifactorial, involving autonomic and enteric neuropathy, depletion of the interstitial cells of Cajal, smooth-muscle dysfunction, and the acute inhibitory effect of hyperglycemia on gastric motility [1]. Type 1 diabetes carries a higher risk of gastroparesis than type 2 diabetes. Most patients follow a chronic course dominated by nausea, vomiting, early satiety, and labile glycemia, whereas life-threatening surgical complications are uncommon. As one of the more common chronic complications of diabetes mellitus, gastroparesis is managed predominantly with supportive measures such as glycemic optimization, dietary modification, prokinetic therapy, and nutritional support, and surgery is reserved for refractory disease or its complications [1,2]

Acute gastric dilatation (AGD) is the pivotal event that can link gastroparesis to catastrophe. Recognized causes include eating disorders and binge eating, mechanical gastric outlet obstruction, and non-mechanical causes such as diabetic gastroparesis, electrolyte disturbances, and critical illness. As intragastric volume and pressure rise, the gastric wall is progressively stretched; when intramural pressure exceeds venous and, ultimately, arterial perfusion pressure, mucosal and then transmural ischemia ensues, culminating in necrosis and perforation [3-6]. Although the stomach is relatively protected by a rich anastomotic blood supply, the fundus and greater curvature are comparatively vulnerable watershed territories, consistent with the fundic necrosis observed in our patient [4,6]. Gastric necrosis from AGD is rare but has been reported to carry a mortality as high as 80-100% [7].

Most published cases of AGD complicated by necrosis or perforation are associated with eating disorders or single massive binge episodes [4,5]; diabetic gastroparesis as the primary driver, documented by sequential imaging, is seldom reported. We present such a case, in which three CT studies captured the entire trajectory from a normal stomach to perforation.

## Case presentation

A 19-year-old woman with insulin-dependent type 1 diabetes mellitus (diagnosed more than five years previously) and a history of asymptomatic cholelithiasis (incidentally documented on abdominal ultrasound earlier that year, with no gastric distension) presented with a four-month history of recurrent admissions for diabetic ketoacidosis (DKA), poor glycemic control, and progressive epigastric pain. Her home medication was insulin glargine 23 units in the morning plus regular insulin as needed. She had no known drug allergies. Glycated hemoglobin (HbA1c) obtained during the current admission was 9.3% (estimated average glucose approximately 220 mg/dL; reference <5.7%, treatment target <7%), while a value obtained approximately 3.5 years earlier had been 13.8% (estimated average glucose approximately 349 mg/dL), indicating chronic, poorly controlled diabetes despite some recent improvement.

**Figure 1:**
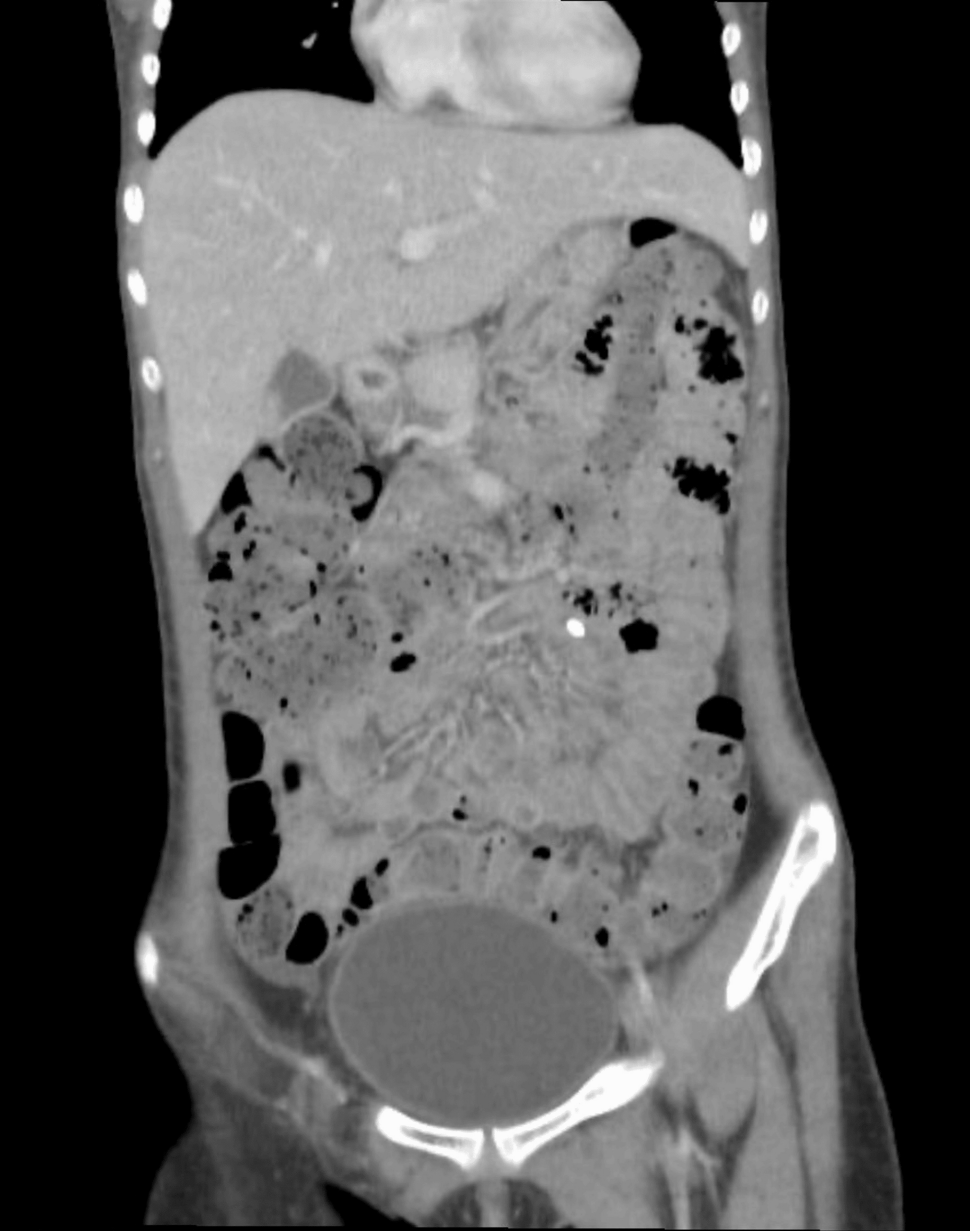
Baseline contrast-enhanced abdominal CT at initial presentation (Day 0) The stomach is of normal caliber, without distension, and no free intraperitoneal fluid is present; incidental hyperdense gallbladder content consistent with microlithiasis was also noted. No arrow is shown, as this baseline study demonstrates no focal abnormality. CT, computed tomography.

**Figure 2:**
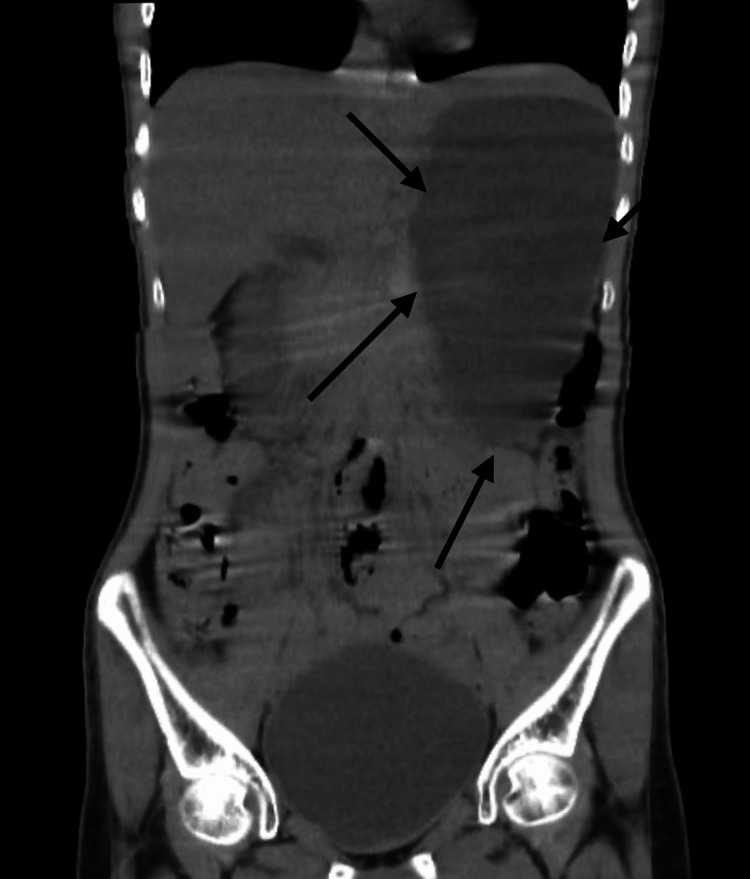
Abdominal CT at 10 weeks after initial presentation showing marked gastric distension The arrows indicate the markedly distended, fluid-filled stomach. An air–fluid level was present in the antral projection, with marked, predominantly hypodense distension of the duodenum (first and second portions); there was no pneumoperitoneum or free fluid. CT, computed tomography.

The clinical and radiological course is summarized in Table 1. At the initial presentation (Day 0), four months prior to the current presentation, she was admitted with abdominal pain, nausea, and vomiting in the setting of DKA, and contrast-enhanced CT of the abdomen showed a nondistended stomach and no acute abnormality, aside from hyperdense gallbladder content consistent with microlithiasis (Figure 1). Approximately eight weeks later, she reported several weeks of epigastric pain, inappetence, and difficulty eating, with imaging then reported as unremarkable. At about 10 weeks, after approximately one month of epigastric pain with vomiting, she was readmitted with DKA. CT of the abdomen at the current presentation demonstrated marked gastric distension with an air-fluid level in the antral projection and marked, predominantly hypodense duodenal distension, without free fluid (Figure 2). At around 13 weeks after the initial presentation, she attended the emergency department for hypoglycemia and was subsequently admitted to the intensive care unit (ICU) for DKA. Throughout these admissions, she was managed for DKA with intravenous fluids, insulin, and correction of electrolyte and acid-base disturbances; CT served as the primary imaging modality, and plain abdominal radiography was not obtained. Notably, dedicated nasogastric decompression was not instituted during the phase of marked gastric distension, as its significance was underappreciated at the time.

**Table 1:** Clinical and radiological timeline Dashes (—) indicate time points at which no imaging was performed. CT, computed tomography; DKA, diabetic ketoacidosis; ICU, intensive care unit; POD, postoperative day.

Time Point	Clinical Event	Imaging Findings
Initial presentation (Day 0)	Admission for abdominal pain, nausea, vomiting; DKA	CT abdomen (contrast-enhanced): non-distended stomach; incidental gallbladder microlithiasis
Week 8	Recurrent epigastric pain (weeks), inappetence, difficulty eating; known cholelithiasis	Imaging reported unremarkable
Week 10	~1 month of epigastric pain with vomiting; admission with DKA	CT abdomen: marked gastric distension with antral air-fluid level and marked hypodense duodenal distension; no free fluid
Week 13	Emergency visit for hypoglycemia	—
Week 14	Admission for DKA (ICU, then ward); discharged home after about one week	—
Week 16 (current presentation; preoperative day)	ICU readmission for DKA complicated by mixed-etiology shock (hypovolemic and septic)	—
Week 16 (day of surgery)	Oral diet resumed after DKA correction; within hours, acute abdominal distension and sudden worsening pain; emergency exploratory laparotomy the same day: partial (fundic) gastrectomy + two-layer gastrorrhaphy (ischemic necrosis of the gastric fundus with multiple perforations, largest 1.5 cm)	CT abdomen: pneumoperitoneum and large-volume free intraperitoneal fluid; bilateral pleural effusions and left basal consolidation
POD 1	Postoperative day 1: ICU, mechanical ventilation, and vasopressor support	—
POD 2–6	ICU postoperative recovery: candidemia (*Candida albicans*, three blood-culture sets) and sepsis managed; extubated and weaned off vasopressors by postoperative day 5; bowel function returned (multiple bowel movements); enteral nutrition tolerated and parenteral nutrition discontinued on postoperative day 6; renal and hepatic function normalized; prognosis favorable	—

**Figure 3:**
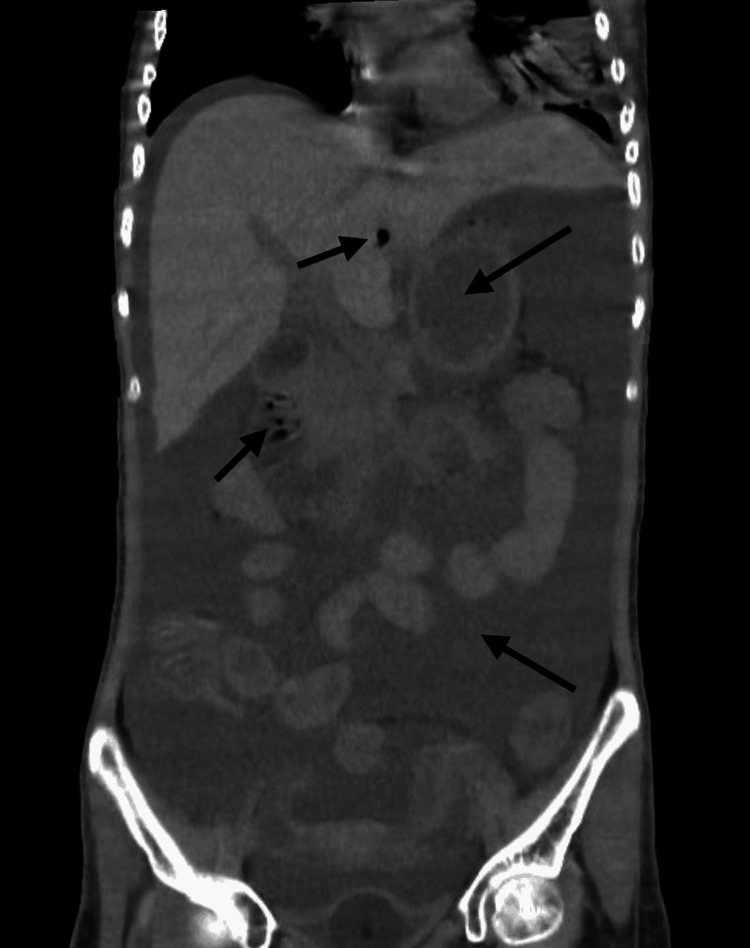
Preoperative abdominal CT on the day of perforation, approximately 16 weeks after the initial presentation, showing pneumoperitoneum and free fluid The arrows indicate foci of free intraperitoneal air (pneumoperitoneum), a discrete sub-diaphragmatic gas bubble and adjacent mottled extraluminal gas locules, the distended gastric chamber, and free intraperitoneal fluid (representative of the large-volume free fluid present). Bilateral pleural effusions (left greater than right) with left basal consolidation were also noted. CT, computed tomography.

At the current presentation, approximately 16 weeks after the initial presentation, the patient was readmitted to the ICU with DKA complicated by mixed-etiology shock (hypovolemia from dehydration combined with septic shock). After correction of the ketoacidosis, an oral diet was resumed the following day; within hours, she developed acute abdominal distension and a sudden increase in abdominal pain that was refractory to optimized analgesia. CT of the abdomen revealed pneumoperitoneum and a large volume of free intraperitoneal fluid, with bilateral pleural effusions (left greater than right) and left basal consolidation (Figure 3). The perforation was confirmed at emergency laparotomy the same day.

On surgical evaluation, she was in poor general condition, sedated, cachectic with marked sarcopenia, pale, and dehydrated. Vital signs were blood pressure 110/70 mmHg, heart rate 110 beats/minute, and respiratory rate 20 breaths/minute. The abdomen was flat, with present bowel sounds, depressible, and diffusely tender with peritoneal signs (peritonism); extremities were warm but poorly perfused, with a capillary refill time of five seconds. The combination of pneumoperitoneum, large-volume free intraperitoneal fluid, and diffuse peritonitis against a background of clinical deterioration mandated emergency exploratory laparotomy for source control. 

Laboratory values across the perioperative period are shown in Table 2. The preoperative arterial blood gas demonstrated severe ketoacidosis (pH 6.87, HCO3 < 3 mmol/L) with profound hyperglycemia (glucose 1002 mg/dL), leukocytosis (23,080/mm³) with 16% band forms, and acute kidney injury (creatinine 2.0 mg/dL). After resuscitation, values transiently improved; the postoperative profile showed recurrent metabolic acidosis (pH 7.18, base excess -14.6 mmol/L), rising C-reactive protein (CRP) (276.2 mg/L), marked left shift (58% bands), aminotransferase elevation (aspartate aminotransferase (AST) 640 U/L, alanine transaminase (ALT) 185 U/L), and severe hypoalbuminemia (albumin 1.8 g/dL).

**Table 2:** Serial perioperative laboratory values Blood gas was arterial preoperatively and on postoperative day 1, and venous on the day of surgery. Preoperative glucose was reported as >500 mg/dL on the gas analyzer and 1002 mg/dL on serum chemistry. Dashes (—) indicate parameters not measured at that time point. ALT, alanine aminotransferase; AST, aspartate aminotransferase; POD, postoperative day; HCO3, bicarbonate

Parameter (Reference range)	Preoperative Values	Day of Surgery	POD 1	POD 2	POD 4	POD 6
pH (7.35–7.45)	6.87	7.40	7.18	7.38	7.40	7.42
pCO2 (35–45 mmHg)	13.0	33.0	34.0	38.0	38	39
pO2 (80–100 mmHg)	121.0	47.0	140.0	125.0	—	135
HCO3 (22–26 mmol/L)	<3	20.4	12.7	22.5	27	25.3
Base excess (-2 to +2 mmol/L)	—	-3.6	-14.6	-2.3	—	—
Sodium (135–145 mmol/L)	124	135	130	145	142	138
Potassium (3.5–5.1 mmol/L)	4.7	4.1	4.4	4.5	3.5	2.5
Ionized calcium (1.10–1.30 mmol/L)	1.10	1.02	1.04	1.19	1.04	—
Glucose (70–99 mg/dL)	1002	283	235	195	—	292
Lactate (0.5–2.2 mmol/L)	2.40	1.3	2.00	1.70	0.9	1.0
Urea (15–45 mg/dL)	79	51	40	—	—	24
Creatinine ([0.6–1.1 mg/dL)	2.0	0.66	0.57	0.18	0.2	0.15
C-reactive protein (<5 mg/L)	<5	44.1	276.2	375.0	327	190
Leukocytes (4,000–11,000 /mm³)	23,080	3,860	4,080	9,800	14,360	19,140
Band forms (0–5 %)	16	10	58	17	—	—
AST (10–40 U/L)	—	72	640	—	—	26
ALT (7–40 U/L)	—	41	185	—	—	32
Phosphorus (2.5–4.5 mg/dL)	—	—	2.5	1.6	2.7	2.3
Albumin (3.5–5.0 g/dL)	—	—	1.8	—	—	—
Magnesium (1.7–2.2 mg/dL)	—	—	1.2	2.2	2.0	1.8
Total calcium (8.5–10.5 mg/dL)	—	—	7.0	7.8	—	—

The patient underwent emergency exploratory laparotomy through an upper midline incision. The cavity contained approximately 3.5 L of gastrobiliopancreatic fluid and food debris, without feculent odor, pus, or blood. Systematic exploration of the small bowel from the ligament of Treitz to the ileocecal valve revealed no lesions; the gallbladder, liver, and spleen were intact. There was ischemic necrosis of the gastric fundus with multiple perforations, the largest approximately 1.5 cm in diameter, with friable tissue and no active bleeding, findings considered consistent with severe diabetic gastroparesis. A partial (fundic) gastrectomy with two-layer gastrorrhaphy (Vicryl 2-0) was performed, followed by copious warm-saline lavage and closure by planes (aponeurosis with Vicryl 1; skin with nylon 3-0). The procedure was completed without intraoperative complications.

The early postoperative course was complicated by sepsis secondary to the gastric perforation and, subsequently, by candidemia (*Candida albicans* isolated from three separate blood-culture sets). Total parenteral nutrition was started; fluid, electrolyte, and acid-base disturbances were corrected; urine output recovered, and antimicrobial therapy was escalated from ampicillin-sulbactam to piperacillin-tazobactam, with fluconazole added for the candidemia.

Over the following days, the patient improved steadily: vasopressors were weaned, she was extubated and was breathing room air by postoperative day 5, and she became alert, oriented, and communicative. Renal function recovered (creatinine 0.15 mg/dL, a low value consistent with her severe sarcopenia and reduced creatinine generation), the transient transaminase elevation resolved (AST 26 U/L, ALT 32 U/L), lactate and acid-base status normalized, and CRP fell from a peak above 300 mg/L to 190 mg/L, although a neutrophilic leukocytosis with left shift (19,140/mm³) and a normocytic, normochromic anemia (hemoglobin 7.0 g/dL, hematocrit 21%; reference hemoglobin 12.0-16.0 g/dL) persisted. Bowel function returned, enteral nutrition was introduced and progressively tolerated, and parenteral nutrition was discontinued on postoperative day 6. Refeeding syndrome was monitored throughout, with ongoing correction of hypokalemia and hypophosphatemia. At the most recent assessment (postoperative day 6), the abdomen was non-tender with a well-healing surgical wound, and the prognosis had become favorable.

## Discussion

Diabetic gastroparesis usually follows a chronic course dominated by nausea, vomiting, early satiety, and labile glycemia, with type 1 diabetes conferring greater risk than type 2. Life-threatening surgical complications are uncommon, which is what makes the present case noteworthy [1,2].

AGD is the pivotal event linking gastroparesis to catastrophe: as intragastric volume and pressure rise, intramural pressure eventually exceeds venous and then arterial perfusion pressure, producing transmural ischemia, necrosis, and perforation, with the fundus and greater curvature representing the most vulnerable watershed zones, consistent with the fundic necrosis seen here [3-6]. This complication is rare but lethal, with reported mortality as high as 80-100% [7].

Most reported cases of AGD with necrosis or perforation are linked to eating disorders or single massive binges [4,5]; diabetic gastroparesis as the primary driver, documented by sequential imaging, is rarely described. In our patient, three CT studies captured the entire trajectory: an initially normal stomach, subsequent massive gastroduodenal dilatation, and finally perforation, providing an unusually complete radiological narrative that is itself instructive.

The relationship between gastroparesis and DKA in this patient was likely bidirectional and self-perpetuating. Hyperglycemia and ketoacidosis acutely impair gastrointestinal motility, while gastroparesis produces erratic nutrient absorption, recurrent vomiting, and poor glycemic control that precipitate further DKA [1,2]. Each ICU admission for DKA may have aggravated gastric atony and promoted progressive dilatation. Across several months, the patient was admitted repeatedly with DKA and recurrent epigastric pain, which was initially attributed to her known cholelithiasis; the significance of the documented gastric distension was underappreciated until AGD and perforation supervened, illustrating how readily this diagnosis is overlooked when an alternative explanation is at hand. Of note, the gallbladder microlithiasis was incidental and asymptomatic, with no clinical or imaging evidence of acute cholecystitis or biliary obstruction; cholecystectomy was therefore not indicated, particularly in a recurrently decompensated, malnourished, and critically ill patient, and at laparotomy, the gallbladder was macroscopically intact.

The marked duodenal distension on the second CT deserves comment. Although compatible with a global motility disorder, it also raises the possibility of a coexisting distal obstructive component, such as superior mesenteric artery (SMA) syndrome [8], to which this severely sarcopenic, cachectic young woman would be predisposed through loss of the aortomesenteric fat pad. Intraoperative inspection from the ligament of Treitz to the ileocecal valve revealed no intrinsic mechanical lesion; however, because SMA syndrome is a position-dependent extrinsic compression that need not leave a visible lesion at laparotomy in a supine, decompressed abdomen, it could not be excluded on that basis and remains a useful differential to consider in similar presentations. Because the study that demonstrated the duodenal distension was acquired without intravenous contrast, the aortomesenteric angle and distance could not be measured at that time, so SMA syndrome can be neither confirmed nor excluded radiologically; accordingly, the diagnosis is best regarded as presumed diabetic gastroparesis with a possible proximal compressive component. A further limitation is that the resected stomach was not submitted for histopathological examination, so the ischemic and necrotic nature of the perforation rests on the macroscopic intraoperative findings rather than on tissue confirmation. Finally, gastric-emptying scintigraphy, the reference standard for diagnosing gastroparesis, was not feasible in this acute, critically ill setting, so the diagnosis of gastroparesis remained clinical-radiological and presumptive. Intraoperative photographs were not obtained in the emergency setting, which precludes pictorial documentation of the operative findings.

Management of severe AGD rests on prompt nasogastric decompression, aggressive correction of fluid, electrolyte, and acid-base derangements, strict glycemic control, and nutritional support, with prokinetics and dietary modification addressing the underlying gastroparesis [1,2]. In the present case, nasogastric decompression was not undertaken during the phase of progressive gastric dilatation because its significance was not recognized at the time; in retrospect, this represented a missed opportunity for earlier decompression and is, in our view, the central teaching point of this report. Once ischemic necrosis or perforation supervenes, urgent operative intervention, the resection of nonviable stomach with repair or partial gastrectomy and thorough peritoneal lavage, is mandatory [3-6], as undertaken here.

The profound hypoalbuminemia (1.8 g/dL) reflected chronic malnutrition and is an adverse prognostic marker for suture-line healing and sepsis resolution [9], underscoring the need for early, structured nutritional support (post-pyloric enteral or parenteral). In the current patient, the perforation became clinically manifest shortly after an oral diet was resumed following correction of DKA, and the early postoperative course was marked by hypophosphatemia, underscoring the risk of refeeding syndrome [10] and the need for cautious, phosphate-aware nutritional reintroduction in chronically malnourished diabetic patients.

Taken together, these findings suggest that chronic diabetic gastroparesis provided the substrate, a dysmotile, recurrently over-distended, and marginally perfused stomach, upon which the resumption of oral intake acted as the proximate trigger for the clinically manifest perforation, rather than gastroparesis producing the acute rupture in isolation. Notably, at her final presentation, the patient was in circulatory shock with peripheral hypoperfusion, reflecting decompensated sepsis and signaling physiologic collapse even before the abdomen was surgically addressed. Her protracted critical illness, central venous access, broad-spectrum antibiotics, and parenteral nutrition predisposed to candidemia [11], a recognized complication in this setting that warrants vigilance and prompt antifungal therapy.

The key clinical lesson, relevant to trainees and experienced clinicians alike, is to maintain a high index of suspicion: in a young patient with diabetes mellitus presenting with persistent vomiting, refractory DKA, and progressive abdominal distension, gastroparesis-related AGD must be considered, and early nasogastric decompression with serial imaging may avert progression to necrosis and perforation.

## Conclusions

Severe diabetic gastroparesis can, although rarely, progress to AGD with ischemic necrosis and perforation, a life-threatening surgical emergency. In this case, serial CT uniquely captured the progression from a normal stomach to massive dilatation and perforation over four months. Early recognition, aggressive glycemic and nutritional optimization, and timely surgical source control are essential to improve outcomes in this rare but highly lethal complication. Despite a catastrophic presentation with septic shock, prompt source control together with structured critical care and nutritional support enabled clinical recovery in this patient. As these observations are derived from a single case, they should be interpreted with caution, and confirmation in larger case series or comparative studies is warranted.
